# Ambulatory Blood Pressure Monitoring in the Elderly

**DOI:** 10.1155/2012/548286

**Published:** 2011-12-20

**Authors:** Juan Diego Mediavilla García, Fernando Jaén Águila, Celia Fernández Torres, Blas Gil Extremera, Juan Jiménez Alonso

**Affiliations:** ^1^Hypertension Unit, Service of Internal Medicine, Virgen de las Nieves University Hospital, 18012 Granada, Spain; ^2^Hypertension Unit, Service of Internal Medicine, Clinic San Cecilio University Hospital, 18012 Granada, Spain

## Abstract

The incidence of hypertension is high in the elderly and is present in 2/3 of the patients older than 65 years. Prevalence can reach 90% in patients older than 80 years. The presence of isolated systolic hypertension (ISH) is characteristic of this population. However, the prevalence of hypertension by ambulatory blood pressure monitoring (ABPM) is not well known. In this study, we analyzed the special characteristics of hypertension in this population, giving special emphasis on ABPM readings.

## 1. Introduction

The incidence of hypertension is high in the elderly, and it is present in 2/3 of the patients older than 65 years [1]. Prevalence can reach 90% in patients older than 80 years [2]. Systolic blood pressure (SBP) increases with age [3], and the presence of isolated systolic hypertension (ISH) is characteristic of this population. However, the prevalence of hypertension diagnosed by ambulatory blood pressure monitoring (ABPM) is not well known. The PROOF study [4] carried out in French patients aged older than 65 years showed that clinical blood pressure (CBP) was elevated in 58% of the patients, and 31% had diurnal SBP >135 mmHg by ambulatory monitoring. In this study, we analyzed the special characteristics of hypertension in this population, giving special emphasis on ABPM readings.

## 2. Characteristics of Hypertension in the Elderly

Ageing results in a decline in the cardiac output and cardiac frequency (beta-receptors-mediated response), as well as a trend towards ventricular hypertrophy, reduction of the left ventricular filling, renal plasma flux and renin plasma, and an increase in renal and peripheral resistances.

Ageing also produces an increase in arterial stiffness, a reduction of the *compliance, *and an increase in the pulse pressure; this, together with an increase in peripheral resistances, leads to ISH. Several systems are involved in the increase of peripheral resistances, such as reduction of beta-2 receptors involved in vasodilation, or reduction in sodium ions, potassium and calcium ions, renin-angiotensin system, sympathetic nervous system, hormones, natriuretic factors, and endothelial factors. All this can explain the frequency of ISH, blood pressure (BP) variability, and the episodes of associated orthostatic hypotension. Old subjects present more frequently essential hypertension (except for renovascular secondary hypertension) and more severe target organ damage than the younger population. Hypertension affects subjects with a higher prevalence of ischemic heart disease, myocardial infarction, diastolic dysfunction, a tendency to arrhythmia and in general cerebral arteriosclerosis and peripheral arterial disease. There are also present other factors such as diabetes, and other concomitant diseases like pulmonary disease, depression, neoplasia, and so forht that should be considered when making diagnosis and treatment decisions.

## 3. Normality Values

Normality values of ABPM are based on several studies [5] (Table 1) on adult populations older than 65 years, despite that it is less represented by subjects aged 70–75 years or older. Published reports of patients older than 65 years showed mean daytime BP values ranging from 128/77 mm Hg [6] to 140/78 mm Hg in a UK study (including ambulatory and hospitalized patients), 134/81 mm Hg in a healthy population [7, 8] in Germany, and values of 138/82 mm Hg were found in patients aged 60–79 years and 147/83 mm Hg in patients older than 80 years [9]. These values could be higher in diabetics or high vascular risk patients.

Therefore, normality values must be carefully studied in patients older than 80 years. It is not known yet the benefit of reducing clinical BP in this population group. Previous studies like HYVET, demonstrated a reduction in the mortality rate in 3.845 patients with SBP >160 mm Hg, with the aim of reaching values of SBP <150 mm Hg, but this study included patients with good physical and mental conditions, without previous cardiovascular disease, and only 7% of them were diabetics.

Bejan-Angoulvant et al. [10] carried out a meta-analysis in patients older than 80 years and demonstrated that intensive treatment reduced 35% of the risk of stroke, 50% of the risk of heart failure, and 27% of cardiovascular events, without differences in total mortality.

Evidences of reducing BP under 140/90 mm Hg in old subjects has been recently discussed; however, any active treatment trial versus placebo therapy has been able to reduce SBP under 140 mm Hg [11]. Therefore, there is no clinical evidence showing which are the normal BP values in old patients and even less by ABPM measurement.

## 4. Isolated Systolic Hypertension

ABPM data of patients with ISH are mainly reported in the SYST-EUR study [12], in patients older than 60 years with baseline clinical systolic blood pressure (CBP) values of 160–219 mm Hg and diastolic BP values lower than 95 mm Hg. Patients were randomized and treated with nitrendipine (10–40 mg/d) and the possible addition of enalapril (5–20 mg/d) according to BP values and/or hydrochlorothiazide (12,5–25 mg/d) and were compared to placebo. SBP was associated with a poorer prognosis.

**Figure 1:**
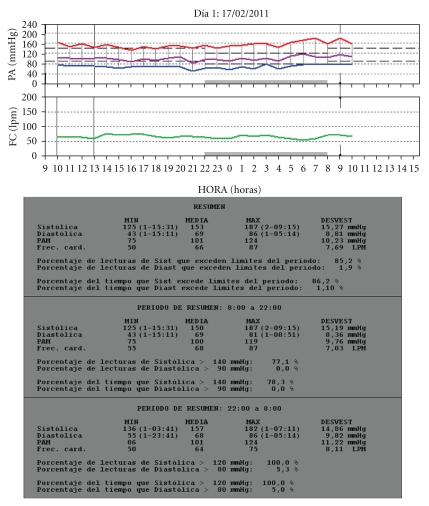
Patient aged 78 years with isolated systolic hypertension and riser pattern.

Clinical SBP of 160 mm Hg was correlated with a 24-h SBP monitoring of 142 mm Hg, 145 mm Hg for daytime BP, and 132 mm Hg for nighttime BP. Differences of the SBP between clinical and ambulatory BP measurement were higher in old subjects (Figure 1).

 A study [13] carried out in 578 patients aged older than 70 years demonstrated not only the predictive capacity of ABPM with respect to cardiovascular morbidity but also the capacity to diagnose hypertensive patients with normal BP values at the doctor's office.

Today, the US and European consensus for ABPM agrees that ABPM measures are important in children and old people, since CBP does not reflect accurately real BP values in these population groups [14]. This is very important to control antihypertensive therapy and establish the most adequate treatment.

## 5. Pulse Pressure and Arterial Distensibility

Both conditions are more frequently found altered in older ages. It is well known that pulse pressure (PP) is an independent cardiovascular risk factor especially present in old subjects [15, 16] as a result of a decline in left ventricular ejection, arterial distensibility, and reflected wave velocity. ABPM is a better estimate of PP than CBP. SYST-EUR analyzed PP by ABPM in 808 patients.

In the placebo group, 24-h and nighttime PP was predictive of total and cardiovascular mortality, stroke, and cardiac events. Daytime PP was a predictor of cardiovascular mortality, all cardiovascular events, and stroke. The hazard rates for 10 mm Hg rise was from 1.25 to 1.68; however, conventional measurement of pulse pressure was 1.35. No significant differences were found in the active treatment group.

Staessen et al. [17] reported that PP estimated by ABPM was a better predictor of events than that measured using CBP in old people with ISH.

In the PIUMA study [18], ABPM values were observed in 2010 untreated hypertensive patients. In this study 24-h PP distribution into terciles was associated with an increase in cardiovascular mortality of 1.19, 1.81, and 4.92 after adjustment for other risk factors. It is suggested that 24-h PP is a good predictor especially when values are higher than 53 mm Hg. Later, in a 3.8-year follow-up study [19] carried out in patients older than 60 years, the highest SBP and PP and lower DBP were correlated with mortality. A median nighttime PP of 78 mm Hg of the 4th quartile showed a 4.4 times greater risk when compared with the first quartile of PP.

However, Masahiro et al., in a study carried out in 1542 Ohasama residents, found that the ambulatory arterial stiffness index (AASI) and PP were reliable to measure arterial stiffness and to predict vascular mortality, although PP when adjusted for age and gender was less efficient in the prognostic value of vascular events of the ABMP or AASI [20].

Arterial distensibility can be measured indirectly by pulse wave velocity, which is altered with age. Asmar et al. [21] found a good correlation between ABPM and arterial distensibility; therefore, ABPM could provide indirectly a reliable assessment of arterial distensibility.

## 6. Nighttime Systolic Blood Pressure

Nighttime SBP is becoming more and more important, as reported in the SYST-EUR study [12] or HOPE substudy [22] in which the effect of nighttime administration of ramipril produced a significant fall (17/8 mm Hg) in nighttime BP values, not appreciated in CBP measurement. In an Anglo-Scandinavian cardiac outcomes trial substudy (ASCOT) [23] carried out in patients with a mean age of 63 years, nighttime SBP was a predictor of vascular events. The decrease in nighttime SBP was higher in the amlodipine-perindopril group than in the atenolol-thiazide group without differences in the daytime BP values. The differences found in the nighttime BP could explain the higher benefit obtained in the amlodipine-perindopril group. DUBLIN study [24] analzed the relationship between CBP, ABPM, and mortality in 1144 patients aged older than 65 years, and found that nighttime SBP was the best correlated, with a risk ratio estimation of 1.18 (1.11–1.25, *P* < 0.001).

## 7. Hypotension

Orthostatic hypotension can occur at any age; however, it is more frequent in the elderly. It is defined as a decrease in the SBP of at least 20 mm Hg or a decrease in the DBP of 10 mm Hg in the orthostatic position 3 minutes after BP measurement in the supine position [25]. It is generally associated with dizziness, slight sweating, and sometimes it is asymptomatic. ABPM is a better predictor of these events than CBP [26]. Many of these events are related to medication (diuretics and beta blockers), diabetes, or disautonomy (baroreceptors) and are more frequently found in the elderly.

**Figure 2:**
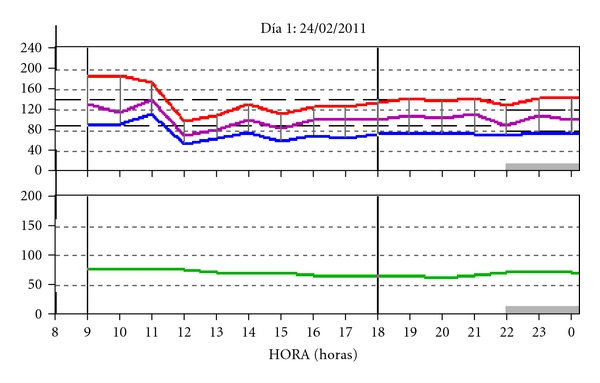
Patient of 79 years old with hypertension and diabetes. Combined therapy with 4 antihypertensive agents. Hypotensive episodes.

Postprandial hypotension is more frequently found in old subjects [27] and is defined as a decrease in SBP of more than 20 mm Hg up to 1 hour after eating, without alteration of heart rate. Postprandial hypotension has been reported as predictor of mortality in some studies [28, 29].  Figure 2 shows the ABPM readings of an old subject with hypotension episodes.

## 8. White-Coat Hypertension

The white-coat effect occurs when CBP is temporary elevated in the clinical setting but not at home. This phenomenon was described by Scipione Riva-Rocci in 1897, but it was really studied in the clinical setting after the introduction of ABPM. Its prognostic significance is not yet clear. In some studies [30–32] mortality rate was 2 or 3 times higher, and it was considered an independent factor for left ventricular mass hypertrophy, increase of arteriosclerosis, or increase in carotid intima-media. Other authors [33, 34] think that white-coat hypertension (WCH) does not correlate with organ damage or cardiovascular events. Age was found to be an independent and common factor influencing WCH in the multivariate analysis of different studies [35–37]. WCH clearly increases with age. In old patients with ISBP [38] the effect of SBP reached 21 mm Hg. These authors reported that the elevation of CSBP in untreated old patients is the main factor to suspect white-coat effect.

## 9. Masked Hypertension

Masked hypertension is defined as a normal blood pressure (BP) in the clinic or office (<140/90 mm Hg), but an elevated BP out of the clinic (ambulatory daytime BP or home BP >135/85 mm Hg) [39]. Its prevalence is not well known, but it has been suggested to be about 10–20% of the general population [40, 41]. It is well known that it is associated with a more severe lesion of the target organ [42–44] and cardiovascular events [13, 45]. It seems that MH is not more frequent in old patients. Bobrie et al. [46] in a study carried out in 4939 patients older than 65 years, reported a prevalence of MH of 9.4%, with CBP <140/90 mm Hg for office BP and 135/85 mm Hg for home BP. In fact, it has been suggested that masked hypertension must be suspected in smoker young men, with unhealthy lifestyle, high vascular risk, diabetics, presence of renal disease with proteinuria, daytime hyperactivity, and patients with transient hypertension [47].

## 10. Circadian Blood Pressure Patterns

ABPM allows knowing BP absolute values and gives information about circadian BP rhythm. According to these patterns, patients are classified as *dipper *(a decrease of BP at night >10%), *nondipper *(BP decrease <10%), *extreme dipper *(BP decrease >20%), and *riser pattern *(an elevation of BP at night). There are increasing evidences that nondipper pattern is associated with a poorer cardiovascular prognosis [48, 49], which has been reported by Staessen et al. [12] in old patients with ISH. Other authors reported that the nondecrease of BP at night is related to the degree of concurrent lesion of the target organ, severity of cardiovascular disease, and alterations in sleep quality [50].

Patients with a lower decrease of BP at night due to insomnia do not seem to present a poorer vascular prognosis, or, if so, it is not related to the nondipping pattern but probably due to a higher value of nocturnal BP [51]. Sleep quality alterations are more frequent in the elderly; therefore, the circadian pattern in these patients must be carried out more carefully. The 24-hour diary to record daily ABPM is indispensable in these patients to adjust the sleep-wake cycle.

A prevalence of 25–35% of the nondipping pattern was observed in the general population. A Spanish study [52] carried out in 42,947 patients showed a nondipping pattern of 41%, which reached 53% in treated hypertensive patients. This pattern can reach up to 60% in high-risk patients [53]. It must be remembered that old patients present higher levels of SBP and a higher vascular risk, target organ damage, diabetes, renal disease, or associated disease, all of them being related to the nondipping pattern [52]. Generally, the loss of the circadian pattern with a lower decrease of nocturnal BP is associated with age, both in men [54] and very old women [7], and in centenarian patients [8, 55].

The morning elevation of BP is also important [42, 56]. Kario et al. [57] in a study carried out in old patients found a clear correlation between lesions in the white substance and the morning elevation of BP, independently of the absolute values of BP.

Further studies are needed in order to determine whether morning elevation of BP is especially relevant in the old population.

Diurnal and nocturnal BP variability (defined by quantification of SD of mean BP) is associated with an increased cardiovascular risk, left ventricular mass, and progression of carotid intima-media thickness [58, 59]. Mediavilla García et al. [60] reported a significant relationship between BP variability, age, and glomerular filtration. In the PIUMA study [61] cardiovascular morbidity was associated with BP variability and age. In a SYST-EUR substudy of 744 old patients, which analzed BP variability in 24 h, daytime, and nighttime, nocturnal BP variability of 5 mm Hg was associated with an increased risk of cardiovascular event of 80% with respect to the placebo group.

## 11. Blood Pressure in Very Old Subjects

Although several studies have reported the relationship between ABPM and cardiovascular risk in the elderly (Table 2), few studies have been published on subjects older than 80 years. Generally these studies [7, 9, 55] are carried out in patient samples with a different population base too small to obtain valid conclusions; however, in this population subset older than 80 years, SBP by ambulatory measurement was higher than that of the subgroup of younger patients. Andrade et al. [62] recently studied 126 patients with a mean age of 83.8 years. The variables associated with the number of cardiovascular events during followup were patients with a clinical history of cerebrovascular event and higher diurnal SBP values.

## 12. High Blood Pressure and Dementia

Dementia from all causes has a prevalence of about 8% of the population over 65 years. Between 15 and 30% of these cases are vascular. In the elderly, subcortical small vessel disease is known to be associated with vascular dementia. Mild cognitive impairment (MCI) is described as a transition phase between healthy cognitive aging and dementia.

This concept facilitated case identification in early stage, and its progression may be preventable through modification of vascular risk factors as hypertension.

The SYST-EUR study [12] showed that a decrease of 7 mm Hg in SBP and 3,2 mm Hg in DBP over 3,9 years will reduce significantly the incidence of dementia. In the Women's Health Initiative Memory Study (WHIMS, [63]), the cognitive function of 7.149 women aged >65 years was assessed using the modified minimental state examination. During a follow-up period of 4,5 years, women with hypertension appeared to be at greater risk of dementia or MCI. Based on these studies, hypertension is associated with the development of MCI and dementia.

ABPM has been shown to provide a better predictive value for cardiovascular events than clinic BP. Recent studies have shown that ambulatory BP variation is associated with cognitive function. High nocturnal SBP level [64], Nondipper status [65], and exaggerated BP variability are suggested to be significant determinants of cognitive impairment. In addition, 24-hour SBP has been shown to be a independent factor for brain atrophy in the elderly [66]. So an strict BP control, including nighttime, may have a neuroprotective effect and prevent the incidence of dementia. The recent literature support that ABPM would help us in an earlier diagnosis of MCI.

## 13. Conclusions

In conclusion, there are many reasons to recommend ABPM in hypertensive old patients, such as ISBP, differences between clinical and ambulatory BP, PP, magnitude of the white-coat effect, assessment of orthostatic hypotension values and its relationship with the patient medication, nighttime hypertension, mild cognitive impairment and BP variability. These important variables demonstrate that just the fact of being an aged patient is a sufficient reason to perform ABPM. We support that the diagnosis of hypertension by ABPM may have substantial clinical and epidemiological implications.

Even so, further studies are needed to demonstrate the importance of ABPM values in the study of the morbidity and mortality in these patients.

## Figures and Tables

**Table 1:** Blood pressure values in adults according to ABPM.

	Optimal values	Normal values	High values
Wake cycle	<130/80	<135/85	>140/90
Sleep cycle	<115/65	<130/70	>135/75

**Table 2:** Studies published on ABPM in subjects older than 65 years.

Study (year)	No. of patients	Age range	Time of followup	Results
Lee et al. (1995) [8]	102	65–93	NA	Normality values
Fotherby and Potter (1995) [7]	108	65–>80	NA	Normotensive values of ABPM Nondipping pattern
Sega et al. (1997) [6]	800	65–74	NA	Population base. Normality values
Hoshide (2002) [67]	811		41 months	ABPM was more useful than CB P in extreme dipper and riser patterns
Wing (2002) [68]	713	65–83	N/A	ABPM prevents overtreatment
O'Sullivan et al. (2003) [9]	156	78	NA	SBP was associated with age Nondipping pattern and age
Björklund et al. (2004) [13]	872 (men)	70	9.5 yrs	Prognosis of cardiovascular risk
Burr et al. (2008) [24]	1144	72	6.7 yrs	ABPM as mortality predictor. Nighttime SBP as best predictor
Ungar et al. (2009) [19]	805	72	3.8 yrs	SBP, PP with low DBP, and mortality
Gosse et al. (2010) [4]	955	>65	N/A	Population base. Prevalence of hypertension
Andrade et al. (2010) [62]	106	83	30 months	ABPM predicts cardiovascular events. SBP loads

NA: not applicable.

## Abbreviations

HORA: Hours
PA: BP
FC: HR
LPM: BPM
Dia 1: Day 1
Horas: Time (hours)
Sistólica: SBP
Diastólica: DBP
PAM: M BP
Frec. Card: HR
MIN: Minimum
MEDIA: Mean
MAX: Maximum
DESVEST SD: Standard deviation
Lpm: bpm (beats per minute)
Porcentaje de lecturas de sist que exceden limites del periodo: Percentage of SBP readings of the period
Porcentaje de lecturas de diastolica que exceden limites del periodo: Percentage of DBP readings of the period
Porcentage del tiempo que diastólica excede limites del period: Time percentage of SBP readings.
